# Blood Neutrophils in Infants Admitted for Bronchiolitis and Subsequent Lung Function Impairment

**DOI:** 10.1002/ppul.71058

**Published:** 2025-07-11

**Authors:** Raffaella Nenna, Laura Petrarca, Maria Giulia Conti, Enrica Mancino, Domenico Paolo La Regina, Francesca Maria Pulcinelli, Alessandra Pierangeli, Enea Bonci, Fabio Midulla, Fernando D. Martinez

**Affiliations:** ^1^ Department of Maternal Infantile and Urological Sciences Sapienza University of Rome Rome Italy; ^2^ Virology Laboratory, Department of Molecular Medicine Sapienza University of Rome Rome Italy; ^3^ The Asthma and Airway Disease Research Center University of Arizona Tucson Arizona USA

To the Editor,

Acute bronchiolitis is a leading cause of morbidity and mortality, particularly in developing countries; it is the most important cause of hospitalization in infants and is often associated with obstructive respiratory sequelae later in life [1]. It remains to be defined if the obstruction is due to an insult caused by the acute respiratory inflammation or it precedes the episode itself. A potential mechanism for airflow limitation postbronchiolitis could be an inappropriate inflammatory response to viral infection [2]. Neutrophils are the dominant cells in the airways of infants with bronchiolitis [3], and in experimental studies in humans, mucosal neutrophilic activation before infection with respiratory syncytial virus (RSV) predicted a symptomatic upper airway disease [4]. Moreover, early after the onset of symptoms in < 6 months infants with severe bronchiolitis, a strong blood neutrophilic response was observed [5]. Whether neutrophilic inflammation is involved in the development of airflow limitation postbronchiolitis is unknown. Defining the role and the characteristics of inflammation in bronchiolitis may identify potentially new therapeutic approaches to these illnesses and new prevention strategies for postbronchiolitis respiratory sequelae.

We hypothesized that increased blood neutrophil counts early during bronchiolitis would be associated with subsequent diminished lung function and respiratory sequelae in the first year after hospital admission.

## 1 Methods

During two consecutive respiratory seasons we enrolled 74 infants (48 in 2021–2022 and 26 in 2022–2023) (age: 2.5 ± 1.0 months, 44.6% males) who were hospitalized for a first episode of bronchiolitis at the Department of Maternal Infantile and Urological Sciences, Sapienza University of Rome, Rome, Italy. Exclusion criteria were children with major congenital abnormalities, immune defects, and lung and airways malformations.

A structured questionnaire was administered to caregivers of all participants regarding gestational and birth information, exposure to cigarette smoke and breastfeeding. A nasopharyngeal aspirate was performed at admission, and samples were tested for 15 respiratory viruses by Reverse Transcriptase‐Polymerase Chain Reaction (RT‐PCR) or nested PCR methods. At admission, as part of standard clinical management, blood cell counts were obtained. Neutrophil counts were adjusted for age and classified as elevated if at or above the median (i.e., 3554 neutrophils/µL) and nonelevated if below the median. During hospitalization a severity score ranging from 0 to 8 was assigned to infants every day. The score at admission and the most severe score were recorded. The score includes four parameters: respiratory frequency (0 = < 45 acts/min; 1 = 45–60 acts/min; 2 = > 60 acts/min), oxygen saturation (0 = > 95%, 1 = 90%–95%; 2 = < 90%), retractions (0 = none, 1 = slight, 2 = severe or nasal flares) and reduction of food intake (0 = normal; 1 = reduced; 2 = fluid therapy required), as previously described. History, vital signs, and clinical course were recorded for each patient. At hospital discharge (T0), at 1 month (mean ages: 4.4 ± 2.1 m) (T1) and 3 months (mean ages: 6.4 ± 1.4 m) (T2) we used an EXHALYZER (EcoMedics, Switzerland) to obtain tidal breathing flow‐volume loops and we calculated time to achieve peak tidal expiratory flow as a percentage of total expiratory time (tPTEF/tE). Pulmonary function tests were performed during quiet sleep, in a supine position with the head midline and the neck slightly extended to minimize upper airway obstruction, using a face mask of size 1 or 2 according to the size and weight of each patient. At 12 months (T3) after discharge, we prospectively followed infants and documented wheezing episodes occurring during the first year with a structured questionnaire, and we defined recurrent wheezing children as those with at least 2 wheezing episodes. The institutional review board and the Policlinico Umberto I approved this study (Prot. 107/12); informed consent was obtained from infants’ caregivers.

## 2 Results

**Figure 1:**
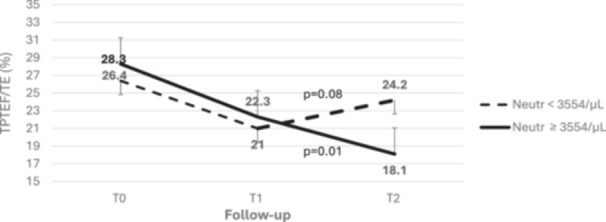
tPTEF/tE by time in children with below the median and at or above the median of age‐adjusted neutrophils at hospital admission for bronchiolitis. Follow‐up: at hospital discharge (T0), at 1 month (T1), and 3 months (T2) after discharge.

Table 1 reports demographic characteristics of enrolled patients. When we divided the 74 infants enrolled according to blood neutrophils count, the severity score at admission and the most severe one were similar in infants with elevated and nonelevated neutrophils. Children with elevated neutrophils counts had similar tPTEF/tE at T0 (26.4% ± 9.0% (*n *= 22) vs. 25.8% ± 9.0% (*n *= 25), *p *= *ns*), and at T1 (*n *= 21, 22.3% ± 6.6% vs. *n* = 26, 21.0% ± 5.3%, *p* = *ns*) and significantly lower at T2 tPTEF/tE (*n *= 37, 20.0% ± 4.3% vs. *n *= 37, 22.9% ± 6.9%, *p* = 0.04) than those with nonelevated counts. Among infants with tPTEF/tE measured at least at T1 and T2 (*n *= 47), lung function was steady during the first month after bronchiolitis and improved at T2 in those with nonelevated neutrophils (from 26.1% ± 9.8% to 21.0% ± 5.3% to 24.2% ± 7.5%, between T0 and T1, *p* =* ns*; between T1 and T2, *p*= 0.08) but significantly decreased (from 28.3% ± 10.6% to 22.3% ± 6.6% to 18.1% ± 3.6%, between T0 and T1, *p* = 0.01; between T1 and T2, *p* = 0.01) among those with elevated neutrophils (Figure 1). As compared with infants with nonelevated neutrophils, those with elevated neutrophils had similar admission and worst severity scores, and similar distribution of viral detection (Table 1). In a multivariate regression, tPTEF/tE at T2 remained significantly associated to neutrophils at admission, after adjustment for sex and the most severe score (*p* = 0.04). When we analyzed the clinical follow‐up, infants with elevated neutrophils counts had more frequently recurrent wheezing by age 1 year (52.8% vs. 26.5%, *p* = 0.03) than those with nonelevated counts. Infants with recurrent wheezing had significantly lower tPTEF/tE than those without, but only at T2 (T0: 25.1% ± 8.1% (*n *= 16) vs. 27.0% ± 9.3% (*n* = 30), *p* = 0.49; T1: 20.4% ± 5.9% (*n *= 16) vs. 22.1% ± 6.1% (*n* = 29), *p* = 0.36; T2: 19.3% ± 4.5% (*n* = 28) vs. 22.5% ± 6.0% (*n* = 42), *p* = 0.017) (see Figure 2, Supporting Information). When we ran a mixed model accounting all the tPTEF/tE calculated at different time points, a steeper slope of tPTEF/tE in infants with elevated neutrophils counts at admission for bronchiolitis was found (test for interaction *p* = 0.06).

**Table 1:** Demographic data and virus detected in the nasal washing of children enrolled.

		Total infants (*n *= 74)	Infants with elevated neutrophils (*n* = 37)	Infants with nonelevated neutrophils (*n* = 37)	*p*
**Age (months)** a		2.5 ± 1.2	2.5 ± 1.2	2.5 ± 1.2	0.96
**Males** ^b^		44.6%	43.2%	45.9%	0.82
**Cesarean section** ^b^		45.2%	47.2%	47.1%	0.73
**Breastfeeding** ^b^		76.7%	73.0%	80.6%	0.44
**Smoking mother** ^b^		17.8%	18.9%	16.7%	0.80
**Smoking cohabitant** ^b^		39.7%	40.5%	38.9%	0.88
**Virus** ^b^	**RSV *n*** = **50**	67.6%	59.5%	75.7%	0.52
	**hRV *n*** = **5**	6.8%	10.8%	2.7%	
	**RSV** + **hRV *n*** = **4 (%)**	5.4%	5.4%	5.4%	
	**Others*n*** = **2 (%)**	2.7%	2.7%	2.7%	
	**Neg *n*** = **13**	17.6%	21.6%	13.5%	
**Score at admission** ^a^		4.0 ± 1.9	4.0 ± 1.9	3.9 ± 2.0	0.85
**Most severe score** ^a^		5.1 ± 1.8	5.3 ± 1.6	5.0 ± 2.0	0.42
**tPTEF/tE T0** ^a^		26.1 ± 8.9	26.4 ± 9.0	25.8 ± 9.0	0.79
**tPTEF/tE T1** ^a^		21.6 ± 5.9	22.3 ± 6.6	21.0 ± 5.3	0.45
**tPTEF/tE T2** ^a^		21.5 ± 5.9	20.0 ± 4.3	22.9 ± 6.9	**0.04**
**Recurrent wheezing**		40.0%	52.8%	26.5%	**0.03**
**tPTEF** ^c^ **/tE T0** ^a^		27.0 ± 10.0	28.3 ± 10.6	26.1 ± 9.8	0.55
**tPTEF** ^c^ **/tE T1** ^a^		21.6 ± 5.9	22.3 ± 6.6	21.0 ± 5.3	0.45
**tPTEF** ^c^ **/tE T2** ^a^		21.5 ± 6.8	18.1 ± 3.6	24.2 ± 7.5	**0.001**

*Note:* Bold values are statistically significant results.

^a^ Number mean ± SD, demonstrated by t‐test

^b^ Number (%), evaluated by Chi–square test.

^c^ Infants with tPTEF/tE at least at T1 and T2.

## 3 Discussion

We demonstrated that infants with elevated peripheral blood neutrophilic responses at hospital admission for bronchiolitis have lung function impairment 3 months after the initial episode, and this was independent of the clinical severity of bronchiolitis and of viral etiology. Moreover, elevated neutrophilic response and reduced lung function were associated with later respiratory sequelae.

As expected, infants after acute bronchiolitis had lower lung function (tPTEF/tE 26.1% ± 8.9%) as compared to 34.8% ± 10.7% that was reported in normal infants [6], and this impairment persisted for at least 3 months after the acute episode (tPTEF/tE 21.5% ± 5.9%) regardless of neutrophil counts at admission. If impairment at discharge is due to pre‐existing airflow limitation or is the direct consequence of the acute episode cannot be determined from our data. However, the novel finding in this study is the further deterioration in lung function as infants moved away from the acute episode among those who had peripheral blood evidence of neutrophilic response early during bronchiolitis. These results suggest that, in this group of patients, whatever their airway size before bronchiolitis, increased neutrophilic responses played a role in further reducing airway caliber, and this process appeared to progress for months after the acute episode.

Several mechanisms have been identified in neutrophil‐mediated inflammation, such as the release of extracellular traps or the production of peptides such as proteases, respiratory oxygen species, and chemokines. We speculate that an exaggerated activation of these neutrophils inflammatory mechanisms may play a detrimental role in the pathogenesis of long‐term respiratory outcomes in infants with bronchiolitis.

## Author Contributions

**Raffaella Nenna:** conceptualization, writing – original draft, writing – review and editing, investigation, methodology, funding acquisition. **Laura Petrarca:** validation, investigation. **Maria Giulia Conti:** investigation. **Enrica Mancino:** investigation. **Domenico Paolo La Regina:** investigation. **Francesca Maria Pulcinelli:** investigation. **Alessandra Pierangeli:** investigation. **Enea Bonci:** data curation, formal analysis. **Fabio Midulla:** conceptualization, supervision. **Fernando D. Martinez:** supervision, conceptualization, writing – review and editing, validation.

## Conflicts of Interest

The authors declare no conflicts of interest.

## Supporting information

Supporting information.

Supporting information.

## Data Availability

The data that support the findings of this study are available from the corresponding author upon reasonable request.
