# Supplementary figures and images for: Blood Neutrophils in Infants Admitted for Bronchiolitis and Subsequent Lung Function Impairment
Source: Pediatr Pulmonol. 2025 Jul 11;60(7):e71058. doi: 10.1002/ppul.71058 (PMC12247150; doi:10.1002/ppul.71058)

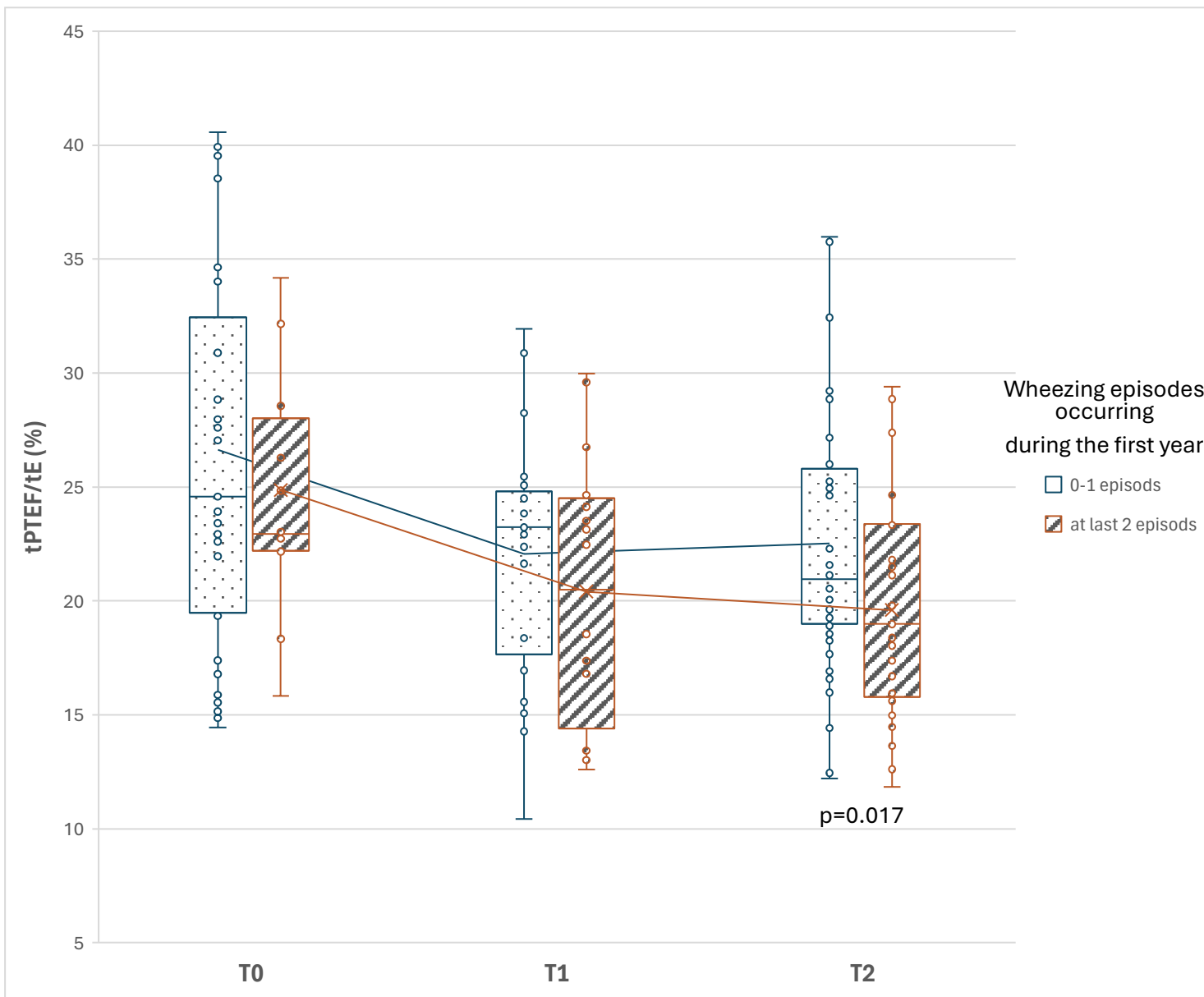

Supplement: Supplementary file 1 — Supporting information. [file PPUL-60-0-s002.pdf]
