# Supplementary material for: Blood Neutrophils in Infants Admitted for Bronchiolitis and Subsequent Lung Function Impairment
Source: Pediatr Pulmonol. 2025 Jul 11;60(7):e71058. doi: 10.1002/ppul.71058 (PMC12247150; doi:10.1002/ppul.71058)
Supplement: Supplementary file 2 — Supporting information. [file PPUL-60-0-s001.docx]

**Online Supplement**

**Blood Neutrophils In Infants Admitted For Bronchiolitis And Subsequent Lung Function Impairment**
^1^Raffaella Nenna, PhD, ^1^Laura Petrarca, PhD, ^1^Maria Giulia Conti, PhD, ^1^Enrica Mancino, PhD, ^1^Domenico Paolo La Regina, MD, ^1^Francesca Maria Pulcinelli, ^2^Alessandra Pierangeli, PhD, ^1^Enea Bonci, BSc, ^1^Fabio Midulla, PhD*, ^3^Fernando D. Martinez, MD*

^1^Department of Maternal Infantile and Urological Sciences, Sapienza University of Rome, Rome, Italy

^2^Virology Laboratory, Department of Molecular Medicine, Sapienza University of Rome, Rome, Italy,

^3^The Asthma and Airway Disease Research Center, University of Arizona, Tucson, USA

*Contributed equally.

**Corresponding Author:**

Raffaella Nenna, PhD ORCID: 0000-0001-8880-3462

Department of Maternal Infantile and Urological Sciences - Sapienza University of Rome

Viale Regina Elena 324 - 00161 Rome

Tel +39 06 49979375; Fax +39 06 49979363

[raffaella.nenna@uniroma1.it](mailto:raffaella.nenna@uniroma1.it)

**Additional references**

1. Openshaw PJ, Yamaguchi Y, Tregoning JS. Childhood infections, the developing immune system, and the origins of asthma. J Allergy Clin Immunol. 2004 Dec;114(6):1275-7. doi: 10.1016/j.jaci.2004.08.024.
2. Martinez FD. Bending the Twig Does the Tree Incline: Lung Function after Lower Respiratory Tract Illness in Infancy. Am J Respir Crit Care Med. 2017 Jan 15;195(2):154-155. doi: 10.1164/rccm.201611-2325ED.
3. Nenna R, Fedele G, Frassanito A, Petrarca L, Di Mattia G, Pierangeli A, Scagnolari C, Papoff P, Schiavoni I, Leone P, Moretti C, Midulla F. Increased T-helper Cell 2 Response in Infants With Respiratory Syncytial Virus Bronchiolitis Hospitalized Outside Epidemic Peak. Pediatr Infect Dis J. 2020 Jan;39(1):61-67. doi: 10.1097/INF.0000000000002505.
4. Frey U, Stocks J, Coates A, Sly P, Bates J. Specifications for equipment used for infant pulmonary function testing. ERS/ATS Task Force on Standards for Infant Respiratory Function Testing. European Respiratory Society/ American Thoracic Society. Eur Respir J. 2000 Oct;16(4):731-40. doi: 10.1034/j.1399-3003.2000.16d28.x.
5. Qi YY, Jiang GL, Wang LB, Wan CZ, Zhang XB, Qian LL. Lung Function in Wheezing Infants after Acute Lower Respiratory Tract Infection and Its Association with Respiratory Outcome. Chin Med J (Engl). 2017 5th Jan 2017;130(1):4-10. doi: 10.4103/0366-6999.196577.
6. Sebina I, Phipps S. The Contribution of Neutrophils to the Pathogenesis of RSV Bronchiolitis. Viruses. 2020 Jul 27;12(8):808. doi: 10.3390/v12080808.

**Figure 2: Box plot of tPTEF/tE by the presence of recurrent wheezing by age 1 year.**

**Legend**: Follow-up: at hospital discharge (T0), at one month (T1) and 3 months (T2) after discharge.
